# Deep Learning–Driven Glaucoma Medication Bottle Recognition: A Multilingual Clinical Validation Study in Patients with Impaired Vision

**DOI:** 10.1016/j.xops.2025.100758

**Published:** 2025-03-07

**Authors:** Aidin C. Spina, Christopher D. Yang, Ayush Jain, Christine Ha, Lauren E. Chen, Philina Yee, Ken Y. Lin

**Affiliations:** 1 School of Medicine, University of California, Irvine, California; 2 Department of Ophthalmology, Gavin Herbert Eye Institute, University of California, Irvine, California; 3 Department of Computer Science, University of California, Irvine, California; 4 Department of Biomedical Engineering, University of California, Irvine, California

**Keywords:** Clinical validation, Artificial intelligence, Convolutional neural network, Glaucoma medication, Medication compliance

## Abstract

**Objective:**

To clinically validate a convolutional neural network (CNN)-based Android smartphone app in the identification of topical glaucoma medications for patients with glaucoma and impaired vision.

**Design:**

Nonrandomized prospective crossover study.

**Participants:**

The study population included a total of 20 non-English-speaking (11 Spanish and 9 Vietnamese) and 21 English-speaking patients who presented to an academic glaucoma clinic from December 2023 through September 2024. Patients with poor vision were selected on the basis of visual acuity (VA) of 20/70 or worse in 1 eye as per the California Department of Motor Vehicles' driver's license screening standard.

**Intervention:**

Enrolled subjects participated in a medication identification activity in which they identified a set of 6 topical glaucoma medications presented in a randomized order. Subjects first identified half of the medications without the CNN-based app. They then identified the remaining half of the medications with the app. Responses to a standardized ease-of-use survey were collected before and after using the app.

**Main Outcome Measures:**

Primary quantitative outcomes from the medication identification activity were accuracy and time. Primary qualitative outcomes from the ease-of-use survey were subjective ratings of ease of smartphone app use.

**Results:**

The CNN-based mobile app achieved a mean average precision of 98.8% and recall of 97.2%. Identification accuracy significantly improved from 27.6% without the app to 99.2% with the app across all participants, with no significant change in identification time. This observed improvement in accuracy was similar among non-English-speaking (71.6%) and English-speaking (71.4%) participants. The odds ratio (OR) for identification accuracy with the app was 319.353 (*P* < 0.001), with substantial improvement in both non-English-speaking (OR = 162.779, *P* < 0.001) and English-speaking (no applicable OR given 100% identification accuracy) participants. Survey data indicated that 81% of English speakers and 30% of non-English speakers found the app “very easy” to use, with the overall ease of use strongly associating with improved accuracy.

**Conclusions:**

The CNN-based mobile app significantly improves medication identification accuracy in patients with glaucomatous vision loss without increasing the time to identification. This tool has the potential to enhance adherence in both English- and non-English-speaking populations and offers a practical adjunct to daily medication management for patients with glaucoma and low VA.

**Financial Disclosure(s):**

Proprietary or commercial disclosure may be found in the Footnotes and Disclosures at the end of this article.

The management of glaucoma relies primarily on modulating intraocular pressure (IOP), the most powerful modifiable risk factor, through pharmacologic and procedural interventions.^1^ Topical medications are the cornerstone of glaucoma treatment and target various physiologic pathways to lower IOP.^2^ Yet, patients with glaucomatous vision loss often face challenges in correctly identifying and administering these medications,^3^ which contributes to poor adherence and subtherapeutic dosing.^4^ This phenomenon may be due to a combination of factors.^5^ Prior studies have reported that patients with glaucomatous vision loss may struggle to distinguish among medication bottles as they share similar shapes and sizes.^6^ Other studies have noted that the fine print on topical ophthalmic medications can be difficult to read and may interfere with accurate medication identification.^6^, ^7^, ^8^

Drug manufacturers have attempted to avert these issues by using bottle cap color as an indicator for medication class. Even so, reliance on bottle cap color yields inconsistent medication identification, with several studies showing that patients with glaucoma who find bottle cap color necessary to differentiate between their medications are not able to do so accurately, which can contribute to decreased medication adherence and patient–physician miscommunication when discussing treatment regimens.^9^^,^^10^ Patients with glaucoma who do not speak English exhibit further reduced medication adherence rates due to poor health care and language literacy.^11^^,^^12^ This is linked to poor IOP control, worse disease outcomes, and a heightened risk of irreversible vision loss, which can impair quality of life and increase the risk of ophthalmic comorbidities.^13^^,^^14^ Several studies have shown that medication nonadherence has a direct association with glaucomatous visual field degradation.^15^^,^^16^ There is an evident need to improve the practical application of managing the major modifiable risk factor for irreversible vision loss.

Convolutional neural networks (CNNs) are a type of deep learning architecture commonly applied to image analysis, including medication identification.^17^ With training and optimization, CNNs can accurately classify and differentiate glaucoma medications. We previously developed a smartphone app that integrates a MobileNetV2 CNN trained on images of glaucoma medication bottles that can identify them with high sensitivity and specificity.^18^ A validation study has been published describing its efficacy in patients with glaucomatous vision loss.^19^

The present study aims to improve our previously described CNN model and introduce a streamlined and updated mobile app to the clinical setting for validation. We test a revised CNN trained on a comprehensive dataset consisting of >21 000 images of 8 commonly prescribed glaucoma medications utilizing the YOLOv4 object detection and segmentation architecture.^20^ After training, we integrate our CNN into a mobile app designed for the Android operating system to enable real-time medication identification via smartphone. The objective of the present study is to clinically validate a CNN-based Android app in the identification of topical glaucoma medications for patients with glaucoma and decreased visual acuity (VA).

## Methods

### Participant Eligibility

From December 4, 2023, through September 25, 2024, patients obtaining care from our tertiary care academic glaucoma clinic were screened for study eligibility through the electronic medical record. Adult patients were considered eligible to participate if they had a tested VA of 20/70 or worse in ≥1 eye on the day of their clinic visit, had a glaucoma diagnosis that ranged from glaucoma suspect to severe in stage, and spoke English, Spanish, or Vietnamese. The VA threshold of 20/70 or worse in ≥1 eye was based on the California Department of Motor Vehicles' requirement that anyone applying for a driver's license must have a VA of 20/40 in 1 eye and at least 20/70 in their other eye.^21^ During both segments of the medication identification activity, the better-seeing eye was covered if a participant had a VA better than 20/70 in ≥1 eye. The eye with a VA of 20/70 or worse always remained uncovered. Potential participants were approached privately in an examination room by 2 of the coauthors—A.C.S. and C.H., who are native Spanish and Vietnamese speakers, respectively—and provided with a detailed explanation of the study in their primary language. All included participants gave verbal consent to enroll in the study, which was approved by the Institutional Review Board at the University of California, Irvine. All research adhered to the tenets of the Declaration of Helsinki.

### CNN Training, Dataset Preparation, and Smartphone App Development

Our training dataset consisted of 34 002 images of 8 commonly prescribed topical ophthalmic medications: Alphagan (brimonidine tartrate 0.1%), Combigan (brimonidine tartrate/timolol maleate 0.2%/0.5%), Dorzolamide (dorzolamide HCl ophthalmic solution 2%), Latanoprost (latanoprost 0.005%), Pred Forte (prednisolone acetate 1%), Rhopressa (netarsudil 0.02%), Rocklatan (netarsudil and latanoprost ophthalmic solution 0.02%/0.005%), and Vigamox (moxifloxacin ophthalmic solution 0.5%). These medications were chosen due to their frequent use in the academic glaucoma clinic at the University of California, Irvine.^22^ This dataset consisted of 10 000 original images and 24 002 modified images, generated through various data augmentation techniques to improve model generalizability. Preprocessing steps, applied to all image sets, included auto-orientation to standardize pixel ordering and resizing to ensure uniformity in image size for optimal training. Augmentation techniques included:

1. Orientation flipping: random 180° horizontal or vertical flips to enhance the model's robustness to different bottle orientations.
2. Portrait rotation: random rotations between −20° and 20° to accommodate variation in camera roll.
3. Brightness variation: varying image brightness by ±25% to ensure the model's resilience to different lighting conditions.
4. Gaussian blur: random application of blur to simulate out-of-focus images.

### Data Splitting and Model Training

Our modified dataset was split into 3 subsets: training (88%, n = 21 003), validation (8%, n = 2000), and testing (4%, n = 999). The training dataset was used to train the CNN, whereas the validation dataset was used for hyperparameter tuning and monitoring model performance throughout the training process by calculating metrics such as mean average precision and validation loss. The test dataset served to provide an unbiased evaluation of the model's performance in a production-like environment. The CNN model was based on the YOLOv4 (You Only Look Once Version 4) architecture implemented via the Roboflow 3.0 object detection model. This architecture was selected for its high speed and accuracy in object detection tasks.^20^ Training was performed to optimize model precision and recall, with metrics including mean average precision, precision, and recall used to evaluate model performance.

### Smartphone App Development

**Figure 1:**
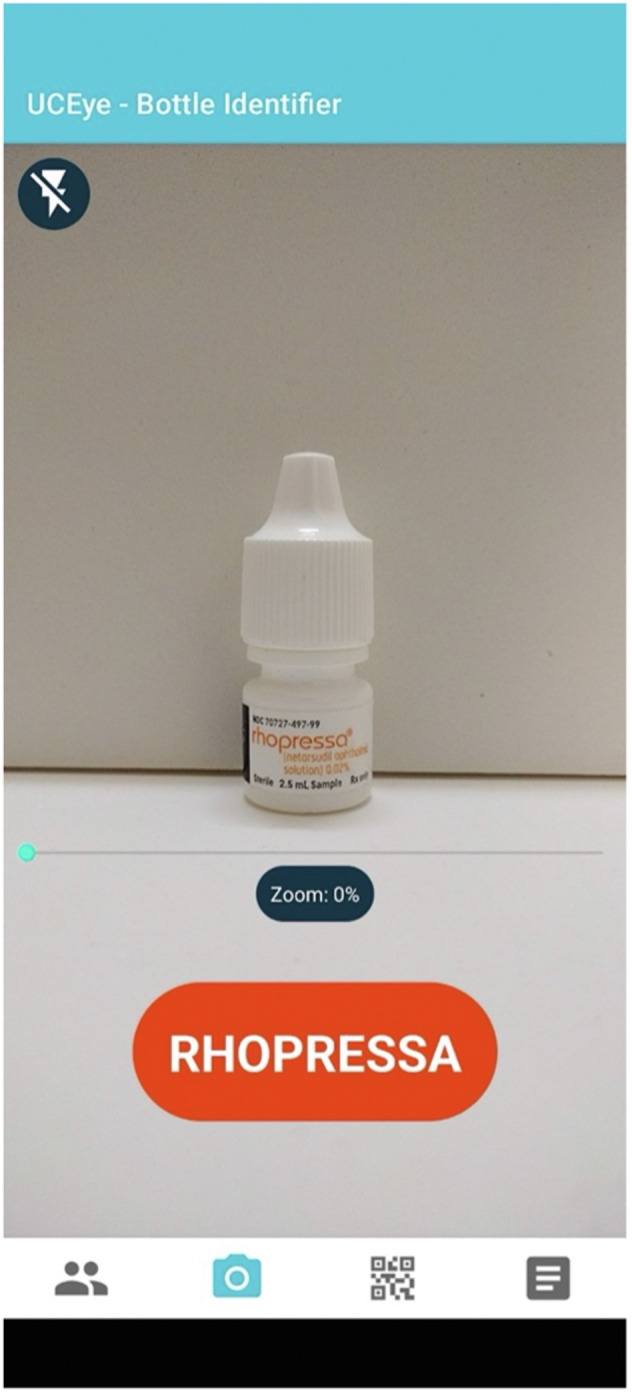
Demonstrated use of UCEye convolutional neural network-based application.

The Android app was designed with 4 primary components: Home Screen, Camera Capture, QR Code Reader, and Prescription Page, which is a library of all medications the user has received from the clinic's QR code, containing information such as medication name, dosage, frequency, and which eye to use it in. Users interact with the app through the Camera Capture page, where they are prompted to point their smartphone camera at a medication bottle. Two recognition modules then activate: a text recognition module first, attempting to identify any medication-specific text, including fuzzy matching for label variations. The app is programmed to utilize the text recognition module first as there is significant variation in glaucoma medication appearance depending on multiple factors, including but not limited to branding and domestic versus international manufacturing. Furthermore, this functionality allows the app to recognize medications beyond the 8 included in the CNN image recognition training, such as timolol. If text identification fails, an image recognition module activates to identify the medication based on visual characteristics. The app includes various accessibility features such as text-to-speech with voice recordings, a pinch zoom/scroll bar, tap-to-focus, and a QR code reader for downloading medication instructions for offline use (Fig 1).

### Clinical Validation

Study participants engaged in a 2-part standardized medication identification activity. Six eye drop medications were used: Alphagan, Combigan, Dorzolamide, Latanoprost, Pred Forte, and Rhopressa. In the first part of the activity, 3 of the 6 medication bottles were randomly selected and presented to the participants in a random order. Six of the 8 eye drops used to train the CNN were utilized in the medication identification activity to avoid disrupting clinic workflow and to keep our methodology consistent with our previously published study.^19^ Participants were asked to identify these medications by name without using the smartphone app. At the beginning of the second part of the activity, all participants were shown how to use the smartphone app using one of the glaucoma medications used in the first part of the activity. The remaining 3 eye drop medications were then shown to the participants in a random order, and the participants were asked to identify the bottles using the smartphone app. A Moto G 5G 2021 Android smartphone (Motorola Inc) was used and set to maximum screen brightness. For participants with VA in both eyes worse than counting fingers, the CNN app's voice function was activated, and the Android smartphone was set to maximum volume. As each eye drop medication has a different font size and label, the order of the 6 eye drop bottles presented to the participants in both parts of the activity was randomized to prevent selection bias.

### Data Collection

Time to medication identification was recorded in both parts of the medication identification activity. Time recording began when the medication bottle was placed on the table in front of the participants and ended when the participants, correctly or incorrectly, made a final identification of the medication. Therefore, each participant produced a total of 6 data points—3 measurements from the first part of the medication identification activity and 3 measurements from the second part of the medication identification activity. At the end of each part of the standardized medication identification activity, all participants were asked to complete a questionnaire to rate their experience identifying medications without and with the smartphone app (Table 1). All participants were asked these survey questions in the primary language indicated on their electronic medical records.

**Table 1:** Participant Questionnaire Post-MIA

Participant Questions Part 1: Without App	Participant Questions Part 2: With App
1. On a scale from 1 to 5 (with 1 being very easy and 5 being very difficult), how easy was it to identify the eye drop bottle? 2. Which features on the bottle are you using to identify the correct medication (e.g., text, color, shape)?	3. On a scale from 1 to 5, how easy was it to use this application? 4. On a scale from 1 to 5, how easy was it to focus the phone camera on the eye drop bottle and take a picture? 5. On a scale from 1 to 5, how easy was it to read the icons and text on the phone screen? 6. Do you think having an app like this would help with your medication-taking process? Why or why not? 7. Did you find the output of the app useful? Are there additional features you would like to be added?

MIA = medication identification activity.

This table shows the questions asked to participants at the conclusion of the MIA. Part 1 assesses participants' ability to identify eye drop bottles based on physical characteristics alone, whereas part 2 evaluates the ease of using the application.

### Medical Chart Review

After preliminary data collection, demographic information, systemic medical history, ophthalmic history (e.g., glaucoma type), and history of prior eye drop use (Alphagan, Combigan, Dorzolamide, Latanoprost, Pred Forte, and Rhopressa) were extracted through each participant's electronic medical record. Glaucoma severity (mild, moderate, or severe) was determined based on the International Statistical Classification of Diseases and Related Health Problems 10 codes diagnosed by the attending glaucoma physician during the participant's clinic visit. A designation of “at risk” was assigned to patients considered glaucoma suspects, whereas a designation of “indeterminate” was given to patients whose stage of glaucoma was difficult to grade due to unreliable field, other conditions that limit vision, or insufficient historic data. Any erroneous data or discrepancies were resolved by discussion and cross-checked for accuracy among chart reviewers.

### Statistical Analysis

Primary outcome measures included identification accuracy, time, and subjective ease of app use. Identification accuracy was assessed across 6 medications used in the medication identification activity. Statistical analysis was conducted using IBM SPSS version 29.0.2.0 (20). The performance metrics of CNN were computed for each medication across training, validation, and test phases. Consultation with a professional statistician from the University of California, Irvine's Biostatistics, Epidemiology, and Research Design unit was conducted to ensure proper statistical methodology and analysis. Statistical analysis consisted of the following: participant demographic analysis, descriptive statistical analysis for CNN performance and primary outcomes (time and accuracy), generalized linear regression analysis to determine the impact of app use on primary outcomes, and chi-square analysis to elucidate the relationship between perceived ease of the medication identification activity and primary outcomes.

## Results

Participant demographics are detailed in Table 2. The mean age of all participants was 59.8 ± 14.9 years. The mean age was 62.25 ± 11.54 years in the non-English-speaking group and 57.43 ± 17.42 years in the English-speaking group. Included participants were 61% male and 39% female. Among non-English-speaking participants, 75% were male and 25% were female, compared with 48% male and 52% female in the English-speaking group. Subgroup analysis of language distribution showed that 55% of non-English-speaking participants (n = 11) spoke Spanish and 45% (n = 9) spoke Vietnamese. The mean VA for all participants was 20/276 in the right eye and 20/417 in the left eye. Non-English-speaking participants had an average VA of 20/283 in the right eye and 20/258 in the left eye, whereas English-speaking participants had an average VA of 20/276 in the right eye and 20/458 in the left eye. A prior history of glaucoma medication use was reported by 83% of all participants, with 90% of English-speaking participants and 75% of non-English-speaking participants having previously used glaucoma medications. Glaucoma severity among participants was categorized as follows: mild (2 non-English-speaking, 0 English-speaking), moderate (2 non-English-speaking, 2 English-speaking), severe (10 non-English-speaking, 17 English-speaking), at risk (3 non-English-speaking, 1 English-speaking), and indeterminate (3 non-English-speaking, 1 English-speaking).

**Table 2:** Patient Demographics

Variable	Non-English Speaking (n = 20)	English Speaking (n = 21)	Combined (n = 41)
Mean age (SD)	62.25 (11.54)	57.43 (17.42)	59.8 (14.9)
Sex (%)	15 male (75%)	10 male (48%)	25 male (61%)
	5 female (25%)	11 female (52%)	16 female (39%)
Language (%)	11 Spanish (55%)	21 English (100%)	11 Spanish (26.8%) 9 Vietnamese (22%) 21 English (51.2%)
	9 Vietnamese (45%)		
Average VA	OD: 20/283	OD: 20/276	OD: 20/276
	OS: 20/258	OS: 20/458	OS: 20/417
Prior glaucoma medication use (%)	15 yes (75%)	19 yes (90%)	34 yes (83%)
	5 no (25%)	2 no (10%)	7 no (17%)
Glaucoma severity (%)			
Mild	2 (10%)	0 (0%)	2 (4.8%)
Moderate	2 (10%)	2 (9.5%)	4 (9.8%)
Severe	10 (50%)	17 (81%)	27 (65.8%)
At risk	3 (15%)	1 (4.8%)	4 (9.8%)
Indeterminate	3 (15%)	1 (4.8%)	4 (9.8%)

OD = right eye; OS = left eye; SD = standard deviation; VA = visual acuity.

This table highlights the pertinent demographic information for the patients who participated in this study.

**Figure 2:**
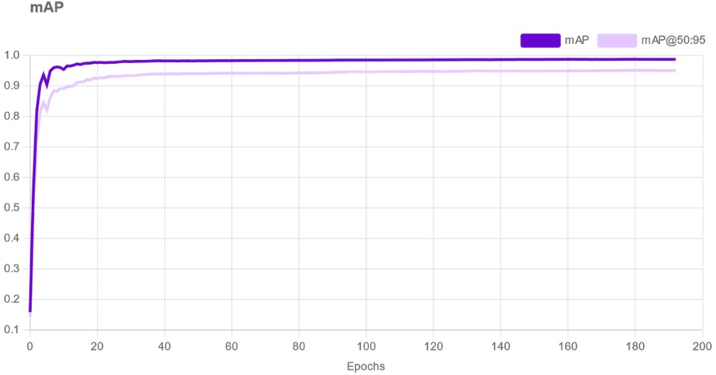
Mean average precision over epochs, i.e. a single iteration in the training process. mAP = mean average precision.

**Figure 3:**
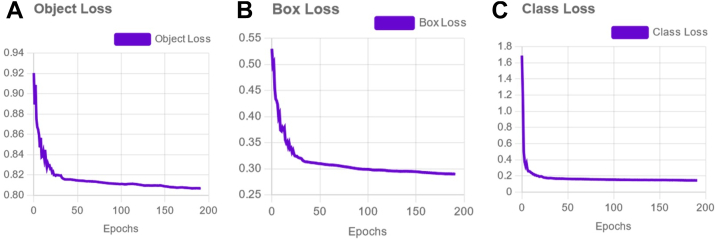
Box (**A**), class (**B**), and object (**C**) losses (over epochs).

The mean average precision achieved by the model across all validation and test datasets was 98.8%, with a recall of 97.2% (Fig 2). Validation and test precision for individual medications in the test set, depicted in Table 3, ranged from 0.94 to 1.0. Additionally, the mean precision for both the validation and test sets was 0.99. Loss metrics—box loss, class loss, and object loss—were tracked over the training epochs, with all loss metrics showing a decreasing trend over epochs, indicating improvement in model training and convergence (Fig 3A–C).

**Table 3:** Medication Identification Precision

Medication	Validation Set Precision	Test Set Precision
Alphagan	0.99	1.0
Combigan	1.0	1.0
Dorzolamide	0.99	0.99
Latanoprost	1.0	1.0
Pred Forte	0.99	1.0
Rhopressa	1.0	0.99
Rocklatan	0.94	0.94
Vigamox	1.0	1.0
All	0.99	0.99

This table lists out all 8 medications that were built into the training set for the application's convolutional neural network along with their validation and test set precision.

In the non-English-speaking group (n = 20), identification accuracy without the app was 26.7% and 98.3% with the app (Table 4). For English-speaking participants (n = 21), accuracy without the app was 28.6% and 100% with the app. The average time to identification in seconds without the app for non-English speakers was 13.7 ± 7.56 and 14.3 ± 9.26 for English speakers. With the app, the average time to identification in seconds was 14.3 ± 8.82 s for non-English speakers and 11.9 ± 4.71 s for English speakers. Combined identification accuracy for all participants was 27.6% without the app and 99.2% with the app. The average time to identification for all participants without the app was 13.99 ± 8.45 s and 13.07 ± 7.09 s with the app. This difference in identification time was not statistically significant (*P* = 0.36).

**Table 4:** Identification Accuracy and Time

Measure	Non-English Speaking	English Speaking	Combined	*P* Value
Accuracy without app (%)	26.7%	28.6%	27.6%	–
Accuracy with app (%)	98.3%	100%	99.2%	–
Average (SD) time in seconds without app	13.7 (7.56)	14.3 (9.27)	13.99 (8.45)	0.91
Average (SD) time in seconds with app	14.3 (58.82)	11.9 (4.71)	13.07 (7.09)	0.16
*t* test comparing time with and without the app *P* value	0.71	0.08	0.36	–

SD = standard deviation.

This table presents a comparison of identification accuracy and the time required for eye drop bottle identification. It shows accuracy rates in both conditions, average identification times in seconds, and statistical comparisons of time using a *t* test where applicable.

Generalized linear regression models were then utilized to determine the statistical relationship between both medication identification accuracy and identification time and mobile app use (Table 5). The odds ratio (OR) for identification accuracy in the combined dataset was 319.35 (95% confidence interval [CI]: 44.578–2287.805, *P* < 0.001). The OR for identification time in the combined dataset was 0.920 (95% CI: −1.640 to 3.480, *P* = 0.481). These findings were consistent with results seen in non-English-speaking participants; in this subset of participants, the OR for identification accuracy with the app was 162.78 (95% CI: 22.407–1182.564, *P* < 0.001), and the OR for identification time was 0.569 (95% CI: −3.110 to 4.248, *P* = 0.762). Within the English-speaking subset, the OR for identification time was 2.34 (95% CI: −5.80 to 1.12, *P* = 0.185). Analysis of identification accuracy was not possible in this subset because 100% of English-speaking patients were able to accurately identify all medications using the app. These findings demonstrate significant improvement in identification accuracy with the app with no significant change in identification time.

**Table 5:** ORs and CIs (Identification Accuracy and Time)

Demographic	Measure	OR	95% CI	Chi-Square	*P* Value
Non-English speaking	Accuracy	162.78	22.407–1182.564	25.332	**<0.001**
	Time	0.569	−3.110 to 4.248	0.92	0.762
English-speaking	Accuracy	–	–	–	–
	Time	2.34	−5.80 to 1.12	1.76	0.185
Combined	Accuracy	319.35	44.578–2287.805	32.944	**<0.001**
	Time	0.920	−1.640 to 3.480	0.496	0.481

CI = confidence interval; OR = odds ratio.

This table presents ORs, CIs (95%), chi-square values, and *P* values for identification accuracy and time based on generalized linear regression analysis. Bold values are considered statistically significant.

Table 6 summarizes responses related to the ease of identifying medications without the app (i.e., question 1) and the ease of using the app to identify medications (i.e., question 3). Responses to question 3 suggest the app was significantly easier to use in the English-speaking group; 81.0% of English speakers found the app very easy to use compared with 30% of non-English speakers. In contrast, 40% of non-English-speaking participants rated the app as slightly difficult to use compared with 9.5% of English-speaking participants. Chi-square analysis was then conducted to compare the ease of medication identification with the app versus without it within each cohort. This analysis for all 3 cohorts (non-English, English, and combined) showed statistically significant differences, with chi-square test statistics of 114.04 (*P* < 0.001), 141.16 (*P* < 0.001), and 126.06 (*P* < 0.001), respectively, highlighting a significant difference in the ease of medication identification both with and without the app, regardless of primary spoken language.

**Table 6:** Chi-Square Analyses with and without the Mobile Application across Non-English Speaking, English-Speaking, and Combined Groups

Response Level	Ease of Identifying the Medication with Your Naked Eye (%)	Ease of Identifying the Medication with the Application (%)	Chi-Square (DOF)	*P* Value
Non-English Speaking				
1 (very easy)	0	30	114.04 (4)	<0.001
2	5	40		
3	25	25		
4	20	0		
5 (very hard)	50	5		
English Speaking				
1 (very easy)	9.5	81	141.16 (4)	<0.001
2	0	9.5		
3	4.8	4.8		
4	19	4.8		
5 (very hard)	66.7	0		
Combined				
1 (very easy)	4.9	56.1	126.06 (4)	<0.001
2	2.4	24.4		
3	14.6	14.6		
4	19.5	2.4		
5 (very hard)	58.5	2.4		

DOF = degree of freedom.

This table presents chi-square analyses comparing the ease of identifying medication with and without the mobile application across non-English speaking (A), English-speaking (B), and combined groups (C).

The relationship between the perceived difficulty of the medication identification activity and identification accuracy and time is summarized in Table 7. Among non-English-speaking participants, the OR for accuracy when comparing difficulty ratings of 4 versus 5 was 9.10 (95% CI: 1.917–43.189, *P* = 0.005), indicating that participants who found the medication identification activity moderately difficult (i.e., difficulty rating 4) were significantly more likely to identify medications accurately compared with those who found it very hard (i.e., difficulty rating 5). In regard to identification time, the OR for difficulty ratings of 3 versus 5 was 4.88 (95% CI: 0.43–9.32, *P* = 0.032). Among English-speaking participants, the OR for identification accuracy when comparing difficulty ratings of 4 versus 5 was 123.0 (95% CI: 11.436–1322.888, *P* < 0.001). Within the same group, the OR for comparing ratings of 3 versus 5 was 82.0 (95% CI: 3.642–1845.997, *P* = 0.006). Across all groups combined, the ORs for ratings of 4 versus 5 and 3 versus 5 were 26.8 (95% CI: 7.730–92.9, *P* < 0.001) and 8.527 (95% CI: 2.29–31.7, *P* = 0.001), respectively. In summary, participants who found the medication identification activity easier (i.e., difficulty ratings 3 or 4) were much more likely to identify medications correctly than those who perceived it as very difficult (i.e., difficulty rating 5).

**Table 7:** Analysis of Ease of Identification and Accuracy

Language Spoken	Comparison	OR	95% CI	Chi-Square	*P* Value
Non-English	Accuracy question 1 (4 vs. 5)	9.10	1.917–43.189	7.724	0.005
	Time and question 1 (3 vs. 5)	4.875	0.431–9.318	4.623	0.032
English	Accuracy question 1 (3 vs. 5)	82.0	3.642–1845.997	7.693	0.006
	Accuracy question 1 (4 vs. 5)	123.0	11.436–1322.888	15.766	<0.001
Combined	Accuracy question 1 (3 vs. 5)	8.527	2.29–31.7	10.238	0.001
	Accuracy question 1 (4 vs. 5)	26.8	7.730–92.9	26.9	<0.001

CI = confidence interval; OR = odds ratio.

This table presents ORs, CIs, chi-square values, and *P* values for the analysis of impact self-perceived ease of identification on medication identification accuracy.

## Discussion

The present study aimed to validate the efficacy of a novel CNN-based mobile app to assist patients with glaucomatous vision loss in identifying frequently used eye drops in glaucoma management. Previous efforts to improve adherence to glaucoma medications, such as telemedicine reminders and video-based education tools, have shown some success; however, neither of these modalities address decreased medication adherence secondary to low VA.^23^^,^^24^ One study by Gupta et al.^25^ analyzed the efficacy of a bottle neck ring adapter to help visually impaired patients more easily identify eye drops and reported an identification accuracy of 73.3% to 91.7% for distinguishing between the various shapes of ring adapters. In contrast, our participants demonstrated an overall accuracy of 99.2% using our mobile app. Our team previously developed a MobileNet V2-based CNN to efficiently and accurately identify 6 different glaucoma medications using visual input.^18^ Our previously published validation study revealed similar results to our current study, with the app improving identification accuracy with a minimal reduction in identification time; however, the improvement in accuracy was greater in this newer iteration of our app (OR = 319.4) compared with the prior iteration (OR = 4.64).^19^ To our knowledge, no other mobile app exists to aid patients in identifying their topical glaucoma medications with this degree of accuracy.

While prior research indicates that various patient, provider, and environmental factors significantly affect adherence, accurate medication identification remains a critical barrier, particularly for patients with impaired VA.^26^, ^27^, ^28^ Specifically, patients with low VA often struggle to distinguish between similarly shaped bottles and read fine print.^7^^,^^29^ By addressing these challenges with a smartphone app, we aim to provide an accessible and reliable tool to overcome a common barrier to medication adherence in glaucoma management in patients with low VA.

Our results demonstrate a substantial improvement in medication identification accuracy with the app without a significant change in identification time and suggest the app streamlines the identification process without imposing additional time burden on the user (Table 4). These findings suggest that the app may present a practical solution to improve daily glaucoma management and potentially improve outcomes for patients with clinically significant glaucomatous vision loss. Additionally, our demographic analysis demonstrated that both English- and non-English-speaking participants benefited from using the app, demonstrating that language barriers do not hinder the use of the app (Table 4). This improvement in performance is critical, as previous studies have shown that patients with limited English proficiency are more likely to experience medication nonadherence, partly due to difficulties in medication identification.^30^^,^^31^ By providing a tool that allows both English- and non-English-speaking participants to identify glaucoma medications accurately without increasing the time burden, our app could help patients with glaucomatous vision loss regardless of the language they speak.

Participant feedback on ease of use revealed interesting trends. English-speaking participants were more likely to rate the app as “very easy” to use compared with non-English-speaking participants (Table 5). Despite this difference in perceived difficulty, improvement in medication identification accuracy was comparable across groups (Table 4, Table 5, Table 7). The increase in odds across both language groups shows that the app improves patient performance while making the medication identification process more intuitive, regardless of patient language or baseline difficulty. At the end of the medication identification activity, we collected feedback from all participants for potential improvements to our app; the most commonly suggested changes were increasing text size and color contrast and including medication descriptions and reminders for the frequency of medication use, particularly for participants who found the app difficult to use. These suggestions are an active area of research in our group and are being integrated into future versions of our app, along with the development of an iOS version of our app.

Future studies should explore the long-term impact of our app on patient outcomes, such as IOP and glaucoma progression. Our study is limited given it is a single-center investigation with a small and specific participant sample, which may limit the generalizability of our findings. Moreover, although we included both English- and non-English-speaking participants, future studies are needed to assess the app's efficacy in larger and more diverse populations, particularly in non-English-speaking groups. Our CNN was trained on a limited set of glaucoma medications; expanding the training dataset to include more glaucoma medications, such as timolol or other ophthalmic medications for other clinical indications, could expand its use case. Further validation studies in different clinical settings are necessary to determine the utility and scalability of this tool across varied health care environments.

This study demonstrates the efficacy of a CNN-based mobile app in improving glaucoma medication identification accuracy among patients with glaucomatous vision loss, regardless of language proficiency. Both English- and non-English-speaking participants saw a dramatic increase in identification accuracy without a significant change in identification time. The benefit of the app for participants who initially found the medication identification activity difficult highlights its potential to not only enhance the accuracy of medication identification but also increase user confidence and make the identification process more intuitive. These findings suggest that our app could play a critical role in enhancing medication adherence, reducing glaucoma disease progression, and improving overall outcomes in glaucoma management. Future iterations of our app should focus on further optimizing accessibility for non-English-speaking populations to maximize its clinical applicability.

## Declaration of Generative AI and AI-Assisted Technologies in the Writing Process

During the preparation of this work, the authors used ChatGPT 4o to provide feedback on grammar and syntax. After using this tool/service, the authors reviewed and edited the content as needed and take full responsibility for the content of the publication.
